# Breastfeeding duration and associations with prevention of accelerated growth among infants from low-income, racially and ethnically diverse backgrounds

**DOI:** 10.1017/S1368980023002689

**Published:** 2023-12-04

**Authors:** Jigna M Dharod, Christina M Frazier, Jeffery Labban, Maureen M Black

**Affiliations:** 1 Department of Nutrition, School of Health and Human Sciences, 319 College Avenue, University of North Carolina at Greensboro, Greensboro, NC 27412, USA; 2 Office of Research, School of Health and Human Sciences, University of North Carolina at Greensboro, Greensboro, NC, USA; 3 RTI International, Research Triangle Park, NC, USA; 4 Department of Pediatrics, University of Maryland, School of Medicine, Baltimore, MD, USA

**Keywords:** Breastfeeding, Infant growth trajectory, Rapid weight gain, Childhood obesity, Infant feeding practices

## Abstract

**Objective:**

To describe breastfeeding rates from early to late infancy and to examine associations between breastfeeding duration and infant growth, including rapid weight gain (RWG, > 0·67 SD increase in weight-for-age *Z*-score), among infants from low-income, racially and ethnically diverse backgrounds.

**Design:**

A short, prospective cohort study was conducted assessing breastfeeding status at infant ages 2, 4, 6, 9 and 12 months. Infant length and weight measurements were retrieved from electronic health records to calculate weight-for-length *Z*-scores and the rate of weight gain.

**Setting:**

Pediatric clinic in the Southeastern USA.

**Participants:**

Mother-infant dyads (*n* = 256).

**Results:**

Most participants were African American (48 %) or Latina (34 %). Eighty-one per cent were participating in the Special Supplemental Nutrition Program for Women, Infants and Children. Infants were breastfed for a median duration of 4·75 months, with partial more common than exclusive breastfeeding. At 12 months, 28 % of the participants were breastfeeding. Infants breastfed beyond 6 months had significantly lower growth trajectories than infants breastfed for 0–2 months (*β* = 0·045, se = 0·013, *P* = 0·001) or 3–6 months (*β* = 0·054, se = 0·016, *P* = 0·001). Thirty-six per cent of the infants experienced RWG. RWG was more common among infants who were breastfed for 2 months or less than 6+ month breastfed group (relative risk = 1·68, CI_95_ (1·03, 2·74), *P* = 0·03).

**Conclusions:**

Breastfeeding beyond 6 months is associated with the prevention of accelerated growth among infants from low-income, racially and ethnically diverse backgrounds, suggesting progress toward health equity.

Rapid weight gain during infancy, defined as an increase in weight-for-age *Z*-score of >0·67 SD, has been associated with an increased risk for obesity in childhood and beyond^(1–4)^. In addition to prenatal factors, such as maternal overweight/obesity and excess weight gain during pregnancy, the rate of weight gain during infancy is influenced by feeding practices^(5–7)^. The Dietary Guidelines for Americans added infant feeding recommendations in the 2020–2025 revision, including exclusive breastfeeding for the first 6 months and continuing it along with complementary foods for 1 year or longer^(8)^.

Breastfeeding is a biologically active delivery system with interacting components that provide multiple benefits to infants, in addition to nutrition^(9)^. Breastfeeding is associated with a lower likelihood of respiratory infections, ear infections, severe diarrhoea, sudden infant death syndrome and improved neurodevelopment^(8)^. The relation between breastfeeding and childhood obesity has been inconclusive. A recent meta-analysis of 153 studies reported that breastfeeding is associated with a 30 % reduction in children’s obesity risk after controlling for socioeconomic status, suggesting that breastfeeding may be associated with normal growth during infancy^(10)^. Studies have found that irrespective of intensity (exclusive breastfeeding *v*. mixed breastfeeding and formula), duration of breastfeeding from 4 to 6 months is associated with reduced risk for rapid weight gain or accelerated growth by half^(11–13)^. However, there is a lack of information on associations between breastfeeding continuation in the complementary feeding phase of post 6 months and growth trajectory among infants from racially and ethnically diverse backgrounds^(14)^.

Latino and African American children are at increased risk for early rapid weight gain, compared to non-Latino White children^(15,16)^. For instance, Taveras *et al.* found that the rate of overweight/obesity among Latino and African American school-age children was almost double than among non-Latino White children, and the higher obesity in turn was associated with a greater likelihood of rapid weight gain during infancy^(15)^. Similarly, in a longitudinal assessment, higher weight gain among African American *v*. White infants in the first 9 months accounted for 70 % of the difference in obesity at 5 years of age^(16)^. Health disparities, including rapid weight gain and obesity, are also associated with other social risk factors, including living below the poverty line and food insecurity^(17,18)^. In alignment with the call for science and strategies to address inequities in the September 2022 House Conference on Hunger, Nutrition, and Health^(19)^, the study objectives were to describe breastfeeding rates from early to late infancy and to examine associations between breastfeeding duration and infant growth, including rapid weight gain, among infants from low-income, racially and ethnically diverse backgrounds.

## Methods

The study was approved by the University of North Carolina at Greensboro’s Institutional Review Board. In total 276 mother-infant dyads were recruited between August 2019 and November 2021 in the waiting area of a pediatric clinic that serves primarily Medicaid recipients from diverse racial and ethnic families. The study inclusion criteria were (1) singleton birth; (2) full-term (≥ 37 weeks); (3) maternal age ≥ 18 years; and (4) infant age < 2 months. Participation was restricted to English or Spanish speakers, as translator services for languages such as Swahili, Arabic and Karen were unavailable. Infants with health issues, such as cleft palate or congenital abnormalities that might affect breastfeeding were not included.

Trained research assistants approached mothers with young infants and explained the study in detail, i.e. participation involved retrieval of anthropometrics from infant’s health records and interviews on sociodemographic, breastfeeding and related infant feeding practices at 2, 4, 6, 9 and 12 months of infant’s age. Mothers who expressed interest, met eligibility criteria and agreed to participate were asked to provide written informed consent for themselves and their infant. Mothers were also asked for permission to access infants’ anthropometric information from clinic records by signing the Health Insurance and Portability and Accountability Act (HIPPA) form. About 80 % of mothers who met eligibility criteria signed the consent and HIPPA forms.

To estimate the necessary sample size, Monte Carlo simulations of the proposed multilevel growth models were conducted using potential effect size estimates (0·2 to 0·5). Standards for minimum coverage were set at 95 %, estimated bias at less than 5 % and power ≥ 0·8, with attrition rates ranging from 10–20 %. For fixed effects of time and breastfeeding status as low as 0·25, a minimum sample estimated was 200 mother-infant dyads for a power of 0·99 to 0·88. With the clinic’s support, recruitment continued beyond the minimum sample target.

The interviews were conducted by research assistants who were fluent in English and/or Spanish and trained in interviewing skills, study-specific questions and data collection procedures. Infants’ weight and length measurements taken by trained pediatric nurses were retrieved from clinic records. Participants received a grocery gift card at each interview. Before the COVID-19 restrictions in March 2020, all 2-month interviews were conducted in-person at the pediatric clinic, with follow-up interviews conducted either in-person or by phone, depending on participant preference. Beginning March 2020, all interviews were conducted over the phone. To minimise bias and ensure that information was collected in a standardised manner, the principal investigator met with the team weekly to ensure that they were following the protocol and quarterly conducted quality checks, including data review and observations of each research assistant’s interviews.

### Measures

During the interview, information on sociodemographic and breastfeeding status was collected. The interview window was set at +/- 10 days in reference to infant’s age (2, 4, 6, 9 and 12 months of age).

#### Sociodemographic data

During the initial interview at 2 months, mothers reported their race, ethnicity, age, education level, marital status, current body weight and height, and participation in the Special Supplemental Nutrition Program for Women, Infants and Children (WIC) and Supplemental Nutrition Assistance Program. We used their reported weight and height to calculate BMI.

#### Breastfeeding status

Questions from the Infant Feeding Practices Study II, sponsored by the Food and Drug Administration and the Centers for Disease Control and Prevention, were used to determine breastfeeding status at each interview^(20)^. Upon confirming breastfeeding initiation, we asked the following question at the 2-, 4- and 6-month interviews, i.e. ‘Are you currently breastfeeding or feeding your baby pumped milk?’ Upon confirmation, we clarified whether the mother was exclusively or partially feeding breast milk.

At the 9- and 12-month interviews, we asked the breastfeeding question only if breastfeeding was reported in the previous interview. If the participant answered ‘no’ to the question about current breastfeeding, we asked ‘How old was your baby when you completely stopped breastfeeding and/or stopped giving pumped milk?’ The responses to these questions, along with the interview date and infant’s date of birth, were used to calculate the duration of breastfeeding. The interviews were conducted using the online data capture tool REDCap hosted at the University of North Carolina at Greensboro.

#### Anthropometric data

Infants’ weight and length, measured at well-child visits, were retrieved from clinic records. The pediatric nurses had participated in professional trainings on abnormal parameters for weight in children and limitations associated with weighing devices. After checking the anthropometric data for outliers, inaccuracies or questionable information, fifteen cases were dropped. Five additional participants were dropped due to missing more than two measurements and/or interviews. This resulted in the analysis sample size of 256 (92 %) of the 276 recruited mother-infant dyads.

After verification and quality checks, anthropometric data were used to calculate weight-for-length *Z*-scores based on the age- and sex-specific WHO parameters using the following equation^(21)^: *Z* = ((Weight/M)^L^ – 1)/(L * S).

Rapid weight gain was calculated as a change of > 0·67 SD in the weight-for-age *Z*-score between 2 and 12 months of age^(22)^.

#### Statistical analyses

All analyses were conducted using R version 4.2.1^(23)^. Descriptive statistics were used to examine sociodemographic variables and breastfeeding rates at each time point. Breastfeeding duration was dummy coded into 0–2 months, 3–6 months and 6+ months (reference group); these categories were used to examine differences in weight-for-length trajectory *Z*-scores and the risk for rapid weight gain from ages 2 to 12 months using multilevel growth modeling. Infant age was centred at 2 months; intercepts represented the predicted mean weight-for-length *Z*-score at the hypothetical age at first contact. Based on the literature, sociodemographic variables associated with infant growth were controlled in the models, including infant sex, parity, race, ethnicity, maternal BMI, education and type of delivery. Initial inspection of the data suggested that weight-for-length trajectories were nonlinear. Therefore, likelihood ratio tests were conducted to compare model fit among linear, quadratic and cubic growth models, controlling for breastfeeding duration. Once the most appropriate growth form was identified, other covariates and breastfeeding duration-by-age interactions were entered into the model for testing. Statistical significance was set at *P* < 0·05. The finalised model was refit with age centred at 12 months to test for group differences in 12-month growth status by breastfeeding duration category.

Poisson regression was used to test for differences in the relative risk of rapid weight gain by breastfeeding duration category, including adjustment for sociodemographic variables. The robust SE were calculated using sandwich error estimation for R and used to construct 95 % CI.

## Results

As shown in Table 1, 82 % of the participants were either African American (48 %) or Latino (34 %). Over half of the mothers had graduated from high school or earned a GED (56 %). At the 2-month interview, 38 % were employed full-time or part time; 81 % participated in WIC, and 46 % participated in Supplemental Nutrition Assistance Program. Slightly under half (44 %) of the infants were male.

**Table 1:** Sociodemographic characteristics overall and by breastfeeding duration categories (*n* 256)

	Overall		0–2 months* *n* 100 (39 %)		3–6 months *n* 52 (20 %)		6+ months *n* 104 (41 %)		*P* †
	Mean	sd	Mean	sd	Mean	sd	Mean	sd	
Maternal BMI†	31·46	7·30	33·74	8·25	30·90	6·00	29·61	6·00	<0·001
	Median	Range	Median	Range	Median	Range	Median	Range	
Breastfeeding duration (in months)	4·75	0–12·45	0·46	0–2·20	4·11	2·50–6·31	11·73	7·00–12·45	NA
	*n*	%	*n*	%	*n*	%	*n*	%	
Infant sex									**0·807**
Male	112	44	45	40	24	21	43	39	
Female	144	56	55	39	28	19	61	42	
Parity									**0·042**
Primiparous	100	39	45	45	24	24	31	31	
Multiparous	156	61	55	35	28	18	73	49	
Type of delivery									**0·786**
Vaginal	203	79	78	39	43	21	82	41	
C-section	53	21	22	41	9	17	22	42	
Race/ethnicity									**<0·005**
African American	121	48	63	52	22	18	36	30	
Latino	88	34	17	19	21	24	50	57	
Non-Latino White	26	10	15	58	5	19	6	23	
Other‡	21	8	5	24	4	19	12	57	
Education									**0·046**
Less than high school	50	19	14	28	9	18	27	54	
High school/GED	144	56	67	47	29	20	48	33	
Attended college	62	24	19	30	14	23	29	47	
Employment status§									**0·147**
Employed (full/part time)	98	38	45	46	18	18	35	36	
Unemployed	158	62	54	34	34	22	70	44	
Participation in WIC									**0·427**
Yes	208	81	85	41	40	19	83	40	
No	48	19	15	31	12	25	21	44	
Participation in SNAP									**<0·001**
Yes	119	46	60	50	24	20	35	30	
No	137	54	40	30	28	20	69	50	

Percentages rounded. WIC: special supplemental nutrition program for women, infants and children; SNAP: supplemental nutrition assistance program.

* Group of 0–2 months includes 11 % who did not initiate breastfeeding.

† Between-group differences tested using chi-square tests for categorical variables, while for continuous variable, i.e. maternal BMI was analysed using ANOVA with Tukey’s HSD.

‡ ‘Other’ group includes American Indian/Alaska Native, Asian and multiple race/ethnicity.

§ Employment status at enrollment.

Overall, the median breastfeeding duration was 4·75 months. In the 0–2-month category, the median duration was 2 weeks (Table 1), accounting for 11 % who did not initiate breastfeeding. In the 3- to 6-month category, the median duration was approximately 4 months. Among participants who continued breastfeeding for 6+ months, approximately half continued for the entire follow-up period (*median* = 11·73 months; Table 1).

**Fig. 1:**
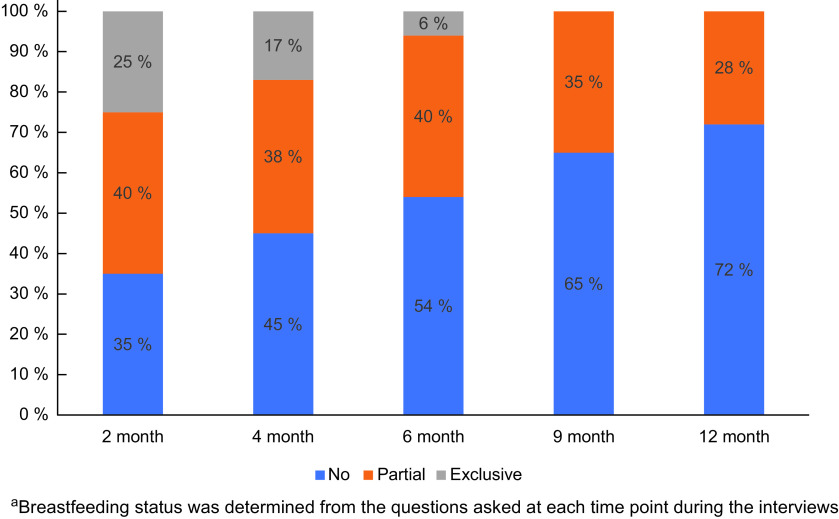
Breastfeeding rates from 2 to 12 months among low-income, racially and ethnically diverse population group (*n* 256)^a^.

As shown in Fig. 1, at each time point of 2, 4 and 6 months, 65 %, 45 % and 46 % of the mothers, respectively, were either partially or exclusively breastfeeding, with partial more common than exclusive. Based on differences in the percentage of women breastfeeding, the overall discontinuation rate was approximately 10 % between interview time points (2, 4, 6, 9 and 12 months), with 28 % of the mothers reported breastfeeding at 12 months.

In comparing breastfeeding groups by sociodemographic characteristics, significant differences were seen by maternal BMI, race, ethnicity, parity, maternal education and Supplemental Nutrition Assistance Program participation (Table 1). Mothers in the 6+ months group had the lowest BMI. Breastfeeding continuation after 6 months was highest among Latino and ‘Other’ groups (Asian, American Indian or mixed ethnicity groups), among multiparous women, among better-educated women (high school or higher) and among non-Supplemental Nutrition Assistance Program participants.

### Weight for length

**Fig. 2:**
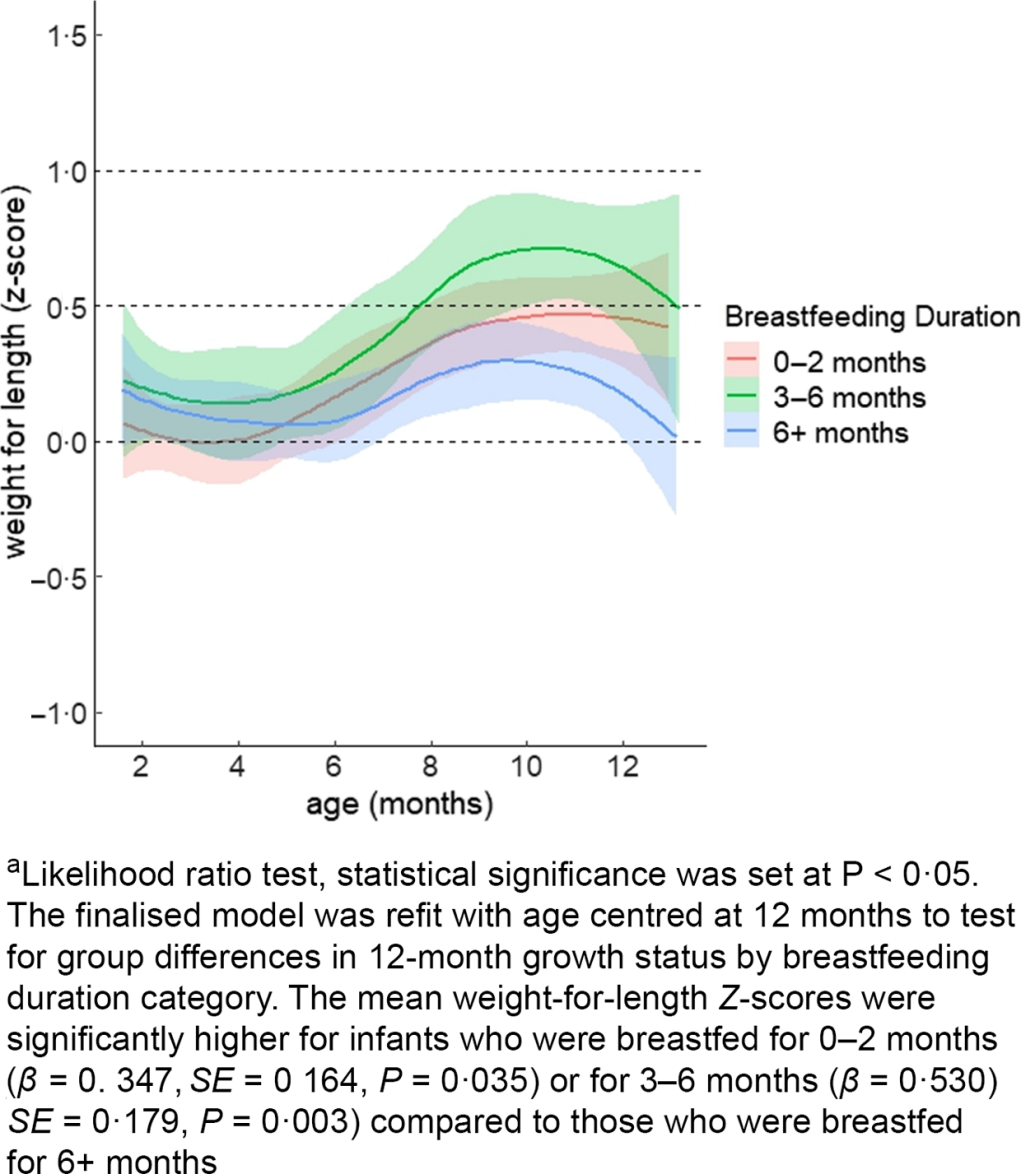
Mean weight-for-length growth trajectory by breastfeeding duration among racially and ethnically diverse group of infants from low-income households (*n* 256)^a^.

The significant interaction of the linear age term with breastfeeding duration indicated that, on average, the per-month change in the weight-for-length *z*-score over the first year of life was greater in the 0–2-month group (*β* = 0·045, *se* = 0·013, *P =* 0·001) or the 3–6-month group (*β* = 0·054, *se* = 0·016, *P =* 0·001) compared to the 6+ month group (Table 2). Consistent with these differences in *Z*-scores, when we refitted the model with time centred at 12 months as shown in Fig. 2, we found that the mean weight-for-length *Z*-scores were significantly higher for infants who were breastfed for 0–2 months (*β* = 0. 347, *se* = 0. 164, *P =* 0·035) or for 3–6 months (*β* = 0·530, *se* = 0·179, *P =* 0·003) compared with those who were breastfed for 6+ months (Fig. 2).

**Table 2:** Multilevel growth model to estimate weight-for-length trajectory by breastfeeding duration (*n* 256)

	*β*	SE	CI_95 %_	*P*
(Intercept)	0·230	0·149	−0·063, 0·523	0·124
Sex	−0·178	0·112	−0·399, 0·042	0·112
African American	0·042	0·193	−0·338, 0·421	0·829
Latino	0·358	0·210	−0·056, 0·772	0·090
Other	0·087	0·265	−0·436, 0·609	0·744
Maternal BMI	0·006	0·008	−0·010, 0·022	0·480
Education-high school/GED	0·126	0·159	−0·317, 0·155	0·430
Education-attended college	0·246	0·189	−0·370, 0·173	0·194
Parity	−0·081	0·120	−0·188, 0·439	0·500
Delivery	−0·098	0·138	−0·126, 0·618	0·475
Age	−0·119	0·044	−0·206, −0·032	0·008
Age^2^	0·036	0·011	0·014, 0·058	0·002
Age^3^	−0·002	0·001	−0·004, −0·001	0·002
BF 0–2 months	−0·044	0·149	−0·338, 0·250	0·770
BF 3–6 months	0·035	0·166	−0·292, 0·362	0·833
Age x BF 0–2 months	0·045	0·013	0·019, 0·072	**0·001**
Age x BF 3–6 months	0·054	0·016	0·023, 0·085	**0·001**

Independent variable breastfeeding (BF) duration, with 6+ month breastfeeding as a referent; Age x BF 0–2 months and Age x BF 3–6 months are differences in mean linear changes over 12 months in weight for length compared to ‘6+ months’ breastfeeding group.

Age^2^ and Age^3^ are quadratic and cubic terms, respectively.

Reference groups for sex: male; race/ethnicity: non-Latino White; education: < high school; parity: primiparous; delivery: vaginal.

### Rapid weight gain

Thirty-six per cent of the infants experienced rapid weight gain between 2 and 12 months. Compared to the 6+ month group, the risk for rapid weight gain was significantly higher in the 0–2-month group (relative risk (RR) = 1·68, CI_95_ (1·03, 2·74), *P* = 0·037) and marginally higher in the 3–6-month group (Table 3). Maternal BMI was positively associated (RR = 1·02, CI_95_ (1·00, 1·05), *P* = 0·007) with rapid weight gain among infants, and having a high school diploma or GED was associated with a decreased risk of rapid weight gain from 2 to 12 months of age compared to having less than a high school education (RR = 0·51, CI_95_ (0·30, 0·85), *P* = 0·011, Table 3).

**Table 3:** Risk ratios for rapid weight gain from 2 to 12 months by breastfeeding duration (*n* 256)

	RR	CI	*P* *
Intercept	0·20	0·11, 0·34	0·000
Maternal BMI	1·02	1·00, 1·05	0·007
Sex (Ref: Male)			
Female	0·79	0·55, 1·13	0·205
Ethnicity (Ref.: non-Latino White)			
African American	1·27	0·66, 2·44	0·464
Latino	1·01	0·48, 2·14	0·964
Other	1·58	0·69, 3·64	0·275
Maternal education (Ref.: < high school)			
High school or GED	0·51	0·30, 0·85	0·011
College education	0·60	0·32, 1·11	0·105
Parity (Ref: Multiparous)			
Primiparous	1·19	0·79, 1·81	0·396
Type of delivery (vaginal)			
C-section	1·32	0·81, 2·15	0·254
Breastfeeding duration (Ref: 6+ months)			
0–2 months	1·68	1·03, 2·74	**0·037**
3–6 months	1·56	0·93, 2·62	**0·092**

RR: relative risk.

* Poisson regression.

Rapid weight gain was defined as a change of > 0·67 SD in weight-for-age *Z*-score between 2 and 12 months; employment status at 12 months of infant’s age.

## Discussion

Our study demonstrates a positive association between continued breastfeeding during the complementary phase and normal growth trajectories among infants from low-income, racial and ethnic groups.

Among our study population, exclusive breastfeeding was relatively uncommon in the first 6 months, and at 12 months, only 28 % of the participants were breastfeeding. At the national level, the overall breastfeeding rate at 12 months is 35 %^(24)^. However, among infants living below the poverty level, the breastfeeding rate at 12 months is 25 %^(24)^, which is consistent with our findings and highlights breastfeeding inequities. Breastfeeding is influenced, not only by individual factors but also by structural and environmental factors, including health care and support services and workplace policies. The relatively low rates of breastfeeding among women living in poverty may be associated with the absence of maternity leave, hourly work positions with limited time for breastfeeding or lack of access to a facility for pumping and storing milk in safe conditions^(25)^. Support through paid maternity leave for all working sectors, including hourly and temporary job workers, has been noted to improve breastfeeding support and in turn, potential expected growth among infants^(26)^. In our study, two-thirds of mothers were not working. Studies have shown that poverty and food insecurity are associated with low breastfeeding self-efficacy and ability to continue breastfeeding^(27,28)^.

National surveys have found that improvements in breastfeeding initiation, exclusivity and continuation have lagged among the African American group compared to other racial and ethnic groups^(24)^. We found the continuation of breastfeeding after 6 months was lower among the African American group, compared to the Latino group. Breastfeeding discontinuation among African American mothers has been associated with younger age, infants with lower birth weight, lack of higher education and low household income^(29)^. A history of structural racism has also played a role by affecting African American women’s trust and confidence in the health care system^(30)^. To address these issues, recommendations include reducing implicit bias and racism among healthcare professionals and improving cultural competency of prenatal and postpartum breastfeeding support programmes^(30)^. Among Latina mothers, relatively higher breastfeeding rates have been attributed to having a family history of breastfeeding, high likelihood of living with a partner and close-knit multigenerational social support^(31)^.

Our study results suggest that breastfeeding throughout infancy is associated with reduced rapid weight gain risk and normal weight status at the end of infancy. Previous studies have also demonstrated this relationship^(11–13)^. For instance, Carling *et al.* ^(12)^ found that children who were breastfed for <2 months were more likely to experience an accelerated growth trajectory compared to children who were breastfed > 4 months. This relationship was particularly significant among children at elevated obesity risk or children whose mothers had a high BMI, low education and/or smoked tobacco during pregnancy. In linking the association between breastfeeding and normal growth, it is possible that breastfeeding may reduce the amount or frequency of formula feeding. Both the nutrient profile of formula and behaviours associated with formula feeding (e.g. finishing the bottle) may increase the risk of rapid weight gain^(32)^. By nutrient profile, the protein content in infant formulas, which is generally higher than in breastmilk, has been associated with higher infant weight gain^(33)^. Additionally, formula contains sweeteners such as maltodextrins and corn syrup, significantly contributing to infants’ daily added sugar intake. In a study with 9- to 12-month-old formula-fed infants, formula contributed to 66 % of the daily added sugar intake and, in turn, was significantly associated with rapid weight gain^(34)^. Among formula-fed infants, practices such as putting an infant to bed with a bottle, adding cereal to bottles and pressuring an infant to finish the bottle, have been shown to increase daily energy intake among infants^(32)^. These behaviours are also applicable to pumped breastmilk feeding. We did not have information on whether infants were breastfed directly or given pumped breastmilk through a bottle. An observation study comparing breastmilk intake directly *v*. pumped milk from a bottle found that a pressurised feeding style, rather than the mode of feeding, predicted milk intake^(35)^. Another study, involving 2553 infants and three feeding modes, found that at 12 months, the formula-fed group had the highest BMI *Z*-scores, followed by the expressed/bottled breastmilk group, and the leanest were the direct breastfeeding group^(36)^.

In the recent past, exclusive breastfeeding rates have increased nationally^(24)^. National programmes, including the baby-friendly hospital initiative^(37)^ and the WIC programme, which serves half of the infants in the U.S.^(37)^, have been critical in not only increasing breastfeeding initiation rates but also advancing policies and programmes to support continued and coordinated breastfeeding services^(37)^. WIC breastfeeding support services and interventions, including text messaging, group education sessions and economic incentives have been effective in improving continuation and intensity of breastfeeding among mothers with limited resources^(38)^. For instance, a randomised controlled trial found that the intervention, which included a monetary incentive in addition to WIC breastfeeding support, was effective in improving breastfeeding continuation up to 6 months^(39)^.

A major strength of our study is the prospective collection of breastfeeding status along with infant growth measurements. Our focus on African American and Latina mothers from low-income settings helps to fill the gap in understanding the protective role of continued breastfeeding among populations experiencing disproportionate burden of childhood obesity. In addition to replication, further research is needed to examine generalizability to other settings as well as whether the positive association between breastfeeding duration and healthy growth persists after 12 months.

There are also several limitations. First, women who were not breastfeeding may have refused to join a study that asked about breastfeeding. Likewise, breastfeeding mothers may have overreported breastfeeding rates, due to perceived social desirability bias. Second, due to limitations of our HIPPA agreement, we did not have access to birth weight and therefore we used 2-month weight status as a baseline, potentially reducing the accuracy of average growth rate. Third, the study lacked the sample size to compare weight gain trajectory by exclusive *v*. mixed breastfeeding and mode of feeding (direct *v*. pumped). In the future, such analysis is warranted to fully understand the associations between breastfeeding patterns and rapid weight gain.

In summary, our study provides information on breastfeeding continuation patterns among racially and ethnically diverse groups of mothers from low-resource settings. Our findings that breastfeeding continuation beyond 6 months is associated with the prevention of rapid weight gain, a precursor of obesity, suggest progress toward health equity.
